# Envisioning multilingualism in source-based writing in L1, L2, and L3: The relation between source use and text quality

**DOI:** 10.3389/fpsyg.2022.914125

**Published:** 2022-07-22

**Authors:** Luan Tuyen Chau, Marielle Leijten, Sarah Bernolet, Lieve Vangehuchten

**Affiliations:** ^1^Department of Management, University of Antwerp, Antwerp, Belgium; ^2^Department of Linguistics, University of Antwerp, Antwerp, Belgium

**Keywords:** source-based writing, source use processes, multilingualism, Inputlog, keylogging, confirmatory factor analysis, structural equation modeling

## Abstract

In this article, we report on a study that investigates how master’s students consult external sources for reading-to-write integrated tasks in their L1 (Dutch), L2 (English), and L3 (French). Two hundred and eighty master’s students in professional communication wrote synthesis texts based on three external sources, including a report, a web text, and a newspaper article in their L1 (Dutch), and in L2 (English), or L3 (French) at two moments of measurement, which were separated by an interval of 6 months. Their source use activities during the writing process were registered using Inputlog – a keylogging program. Inputlog enabled us to determine the amount of time the writers spent composing their main texts and consulting the sources (when the source consultation activities took place during the writing process, which sources were consulted most frequently, and how frequently the writers transitioned between the various sources). Final text quality was assessed holistically using pairwise comparisons (D-pac, now Comproved). Confirmatory factor analysis indicated three components that could describe source use processes in L1, L2, and L3 writing: (a) initial reading time, (b) source interaction, and (c) variance of source use throughout the writing process. Within-subject comparisons revealed that there were no improvements in the students’ text quality in L1, L2, and L3 over an academic year. Structural equation modeling indicated that the source use approach, particularly source interaction, is related to text quality, but only in L1 and L3. We provide further explanations for this variation based on language proficiency, temporal distribution of writing process, and individual differences.

## Introduction

Technological advances have revolutionized the way written texts are generated. In an era marked by the ease of access to information on almost any subject, modern writers often do not start from a blank screen but integrate information from a vast array of sources, such as articles, reports, blogs, tweets, and e-prints to arrive at a final text (Leijten et al., 2014). On the one hand, these newly created synthesis texts must be correct, communicative, clear, coherent, and above all, original, but on the other hand they must retain a certain degree of accuracy and faithfulness to the information represented in the source materials (Leijten et al., 2019). In addition, information in this day and age is also offered in multiple platforms and languages. With a few mouse clicks, modern writers can gain access to information on nearly any topic. Therefore, written texts nowadays, to a large extent, are heavily influenced by external digital sources. Contemporary written communication, in many circumstances, is based on the synthesis of multiple sources into one coherent text.

Writing from multiple sources, or synthesis writing (henceforth used), has become an essential means of communication in both professional and academic contexts. Post-secondary students across the curriculum must write synthesis texts on a regular basis as part of their educational program. However, many students have difficulties incorporating sources into a coherent and communicative text due to the cognitively demanding nature of a reading-to-write task. To begin, a reading-to-write task is significantly different from a writing-only task because synthesis writing combines both reading and writing, as Spivey and King (1989) deemed it “a hybrid task” (Vandermeulen et al., 2020). Indeed, empirical research in writing has shown that reading-to-write tasks demand a particular set of literacy skills and mental processes which transcend those normally found in writing-only tasks (Grabe, 2003; Chan et al., 2014; Chan, 2017; Michel et al., 2020). Consequently, students must juggle reading and writing activities during the writing process: they have to read and understand information in the sources, select only relevant information from the source texts, plan, write, and revise their actual texts. The core element of synthesis writing is to gather information from different sources and structure it around a central theme that serves a certain purpose (e.g., supporting or refuting a claim) (Spivey and King, 1989; Solé et al., 2013; Nelson and King, 2022). Source texts, however, may treat the same subject with multiple perspectives, which could be either complementary or contradictory (Cumming et al., 2016; Leijten et al., 2017; van Weijen et al., 2019). Writers must relate information in source texts to their own knowledge and contrast information across different source texts to select reliable and relevant details and decide what information can be integrated into the target text (Segev-Miller, 2004; Mateos et al., 2014; Martínez et al., 2015). Moreover, synthesis writing involves a dynamic interplay of reading and writing sub-processes, including both macro-skills and micro-skills (Vangehuchten et al., 2018). For example, writers need to distinguish main ideas from supporting details in paragraphs or texts, write solid sentences with correct grammar and spelling, and apply appropriate rhetorical devices to achieve the communicative goals of the text (Alamargot et al., 2007; Asención Delaney, 2008). Finally, a synthesis text requires students to utilize source materials to build disciplinary knowledge of a certain domain, for instance, social sciences, engineering, business, or natural sciences (Weston-Sementelli et al., 2018). To that end, contemporary writing literature acknowledges this reading-to-write process as a socially and cognitively complex activity because writers have to rely on a wide range of linguistic skills and cognitive processes, such as reasoning skills, problem solving skills, and lexical and grammatical resources, on the one hand, and take into consideration psycho-social factors, such as the context, the readership, and the purpose of communication on the other (Spivey and Nelson, 1997; Kellogg, 2008; Plakans, 2008; Ellis, 2009; Schriver, 2012; Byrnes and Manchón, 2014; Lindgren and Sullivan, 2019).

Due to the cognitively demanding nature of a reading-to-write task, it is not surprising that synthesis writing is challenging for L1 writers who write in their own native language (Vandermeulen et al., 2020). Writing from multiple sources in a second or foreign language even places a heavier burden on the cognitive loads and adds another layer to the linguistic and cognitive complexities, as lexical, grammatical, and idiomatic resources tend to be less developed in a second or foreign language (Hinkel, 2003; Doolan and Fitzsimmons-Doolan, 2016). While L1 writers can easily translate their thoughts into writing in a rhetorical manner due to fewer linguistic barriers, L2 writers may experience greater difficulties in retrieving lexical items, adjusting to style, grammar, and spelling, and adapting writing content to satisfy standards of second language writing, especially in academic writing (Grabe and Zhang, 2013). As there seems to be a consensus that L2 learners, especially those in higher education, should be trained to write effectively from sources, synthesis writing has been a productive subject of inquiry in second language writing research (Cumming et al., 2016). However, contemporary intervention studies have failed to reach a one-size-fits-all solution for teaching this specific writing competence (Spivey and Nelson, 1997; Nelson and King, 2022). Unlike spoken discourse, which can be acquired both *via* formal learning and outside the walls of classrooms, learning to write requires long hours of classroom instruction or intensive practice (Cuevas et al., 2016). Because of this, it has been indicated in many studies that education and experience are the common predictive factors for proficiency in synthesis writing (Marzec-Stawiarska, 2016). Without proper training, students’ writing strategies are confined to a linear process of reading sources and the regurgitation of this input into their writing in a relatively straightforward manner, which can eventually lead to patchwork writing or copy-pasting (McGinley, 1992; Lenski and Johns, 1997; Cerdán and Vidal-Abarca, 2008; Plakans and Gebril, 2012; Solé et al., 2013). Such copy-pasting behavior, as well as inappropriate use of sources, may lead to severe academic offenses, namely all kinds of plagiarism. Given the complexities of synthesis writing as a cognitive and social task, many authors have suggested that “textual borrowing” in different forms should be seen as a consequence of lack of training and a pedagogical or learning problem, rather than an intent to cheat, and even more so in L2 than in L1, as it appears to be more common in the latter (Plakans and Gebril, 2013; Neumann et al., 2019; Nguyen and Buckingham, 2019). Despite its increasing importance, recent writing research has revealed that students tend to receive relatively insufficient training on multiple-document synthesis writing (Solé et al., 2013). Therefore, a large body of research has been devoted to helping students integrate sources into writing in an appropriate and ethical manner (Hyland, 2001; Hirvela and Du, 2013; Liu et al., 2016).

Prior research on synthesis writing revolved around various dimensions of synthesis writing. In general, four primary strands of research can be distinguished from the current writing literature: (a) studies that focused on the products (text quality), (b) studies that focused on the process (how writers construct their texts), (c) studies that focused on the perception of writers toward writing activities, and (d) intervention studies (Vandermeulen et al., 2019). Despite variation in design and research dimension, the overarching aim of investigating the ability to write from multiple sources is to understand the underlying cognitive processes of synthesis writing that could lead to well-written texts. Based on the current literature, it remains an open debate as to why some writers tend to be more successful than other writers. To design effective instruction and feedback for synthesis writing process, we need to gain insight into the synthesis writing processes, including source use processes and text-productive strategies, which underlie a high-quality synthesis text.

One factor of particular interest in synthesis writing research is the role of language proficiency in determining writers’ strategies and approach for integrating source information into their synthesis texts. Two opposing theories have been proposed to account for the influence of linguistic proficiency on writers’ source integration strategies: the Inhibition Hypothesis by Schoonen et al. (2009) and the Linguistic Interdependence Hypothesis by Cummins (2016). According to Schoonen et al. (2009), the cognitive loads required for retrieving proficiency-related variables, such as lexical knowledge or grammatical accuracy, are cognitively demanding, so these factors may interfere with synthesis writing processes as they monopolize the cognitive processes (e.g., attention) that could possibly be paid to more global aspects of writing (e.g., idea development, revision). In contrast, Cummins (2016) explains *via* the Linguistic Interdependence Hypothesis that “two languages are distinct but are supported by shared concepts and knowledge derived from learning, experience, and the cognitive and language abilities of learners” (Chuang et al., 2012, p. 98). In this sense, L1 and L2 writers may share many similar behaviors when composing synthesis texts, despite their differences in linguistic profiles. Empirical research in the literature has supported both hypotheses, with several studies indicating the influence of language proficiency on the quality of synthesis written texts. Keck (2006) reported that second language writers tended to use considerably more “near copies” in their summary paraphrasing tasks than did their native English counterparts. In a similar vein, second language learners were found to experience greater difficulties formulating appropriate task representations and developing strategies for writing than did native English writers (Connor and Carrell, 1993; Wolfersberger, 2013). In addition, second language research also confirmed the benefits of language proficiency on writing processes. For example, Plakans and Gebril (2012) reported in their study that students with lower level of English language proficiency experienced difficulties understanding information in source texts and focused mainly on micro-levels of writing, such as vocabulary and grammar, while students with higher language proficiency focused on cohesion, content, and rhetoric. McDonough et al. (2014) found that low-proficiency students had problems with locating main ideas in source texts and reformulating the ideas in source text sentences, which led them to use a compensatory strategy of frequently using short, copied strings from the source materials in their writing. One advantage that writers with high language proficiency have over their low proficiency counterparts may be related to lexical knowledge and the ability to meta-linguistically manipulate words *via* their more developed semantic networks (Baba, 2009), although lexical diversity in L2 writing may also be predicted by lexical diversity in source texts, rather than the individual’s vocabulary load (Gebril and Plakans, 2016). These findings have led Kim (2001) and Asención Delaney (2008) to conclude that cognitively demanding writing tasks might affect students’ performance in accordance with their levels of proficiency in English. On the other hand, Hyland (2009) analyzed a corpus of written compositions and found minor differences between first and second language writers in their referencing practices. Because of this, she concluded, like Keck (2014), that regardless of their linguistic profile, all students seem to undergo similar and predictable stages in their development of coherent citation practices and discourse, which makes it difficult to distinguish strategies and processes that are exclusive to reading and writing in a second language. Due to variation in sampling, measurements of testing, and methodology, these findings remain inconclusive. This has led Cumming et al. (2016) to state in their review article: “The universal nature and challenge of learning to write effectively from sources make it difficult to draw absolute distinction between the writing of L1 and L2 students, particularly given the small numbers of student populations and writing tasks examined to date.”

As writers may encounter even more linguistic and cognitive barriers when writing in L3 than in their L1 and L2, we could measure the extent to which linguistic proficiency may influence the synthesis writing process by comparing L3 writing processes with those of L1 and L2 writing. In comparison with first and second language writing studies, writing research in a third language is relatively limited. Traditionally, the main concern in the field of second language acquisition (SLA) has predominantly focused on how any non-native languages acquired after the first language are learned, and classical SLA researchers have treated second language and third language as similar concepts (Hammarberg, 2001; De Angelis, 2007). However, many recent studies have indicated that the acquisition of L3 may be extremely different from that of L2 since L3 learners study a new language with a native language and another non-native language (which they have been exposed to for a long time and in which they have reached a fairly high level of fluency) existing simultaneously in their mind, which can either complement or contradict each other (e.g., Leung, 2003; Flynn et al., 2004; Bardel and Falk, 2007; Jin, 2009; Rothman and Cabrelli Amaro, 2010; Montrul et al., 2011; Berkes and Flynn, 2012; Hermas, 2014; Rothman, 2015; Slabakova and Garcia Mayo, 2015). Consequently, writing in L3 may have a different impact on the writers as they are influenced by both L1 and L2 in the writing process. In this study, following the definition provided by Reichelt et al. (2012) (as cited in Allen, 2018, p. 516), we consider French to be the L3, since for our subjects these languages are neither the user’s L1 (Dutch) nor the dominant L2 (English) of the surrounding context. Indeed, the participants in this study are Flemish students coming from a master’s program in Multilingual Professional Communication at a Flemish university. In their study program, some of the participants took some courses in English (L2), and others took courses in French (L3). Although French is considered the first foreign language taught in Flanders as of the age of 10, Flemish children are extensively incidentally exposed to English (e.g., television programs, computer games, music) long before they get their first English and French class in school (Simon and Van Herreweghe, 2018). As a result, we consider English as the dominant L2 of the surrounding context and French as the master students’ L3, as its acquisition process mainly takes place in an intentional and scholar setting, with limited presence in the Flemish society as opposed to English. To date, L3 research has predominantly focused on cross-linguistic transfer in relation to low-level components of language, such as the lexicon (Dewaele, 1998; Cenoz et al., 2003; Williams and Bjorn, 2009; Hermas, 2014) or syntax (Flynn et al., 2004; Leung, 2005, 2007; Bardel and Falk, 2007; Westergaard et al., 2017). Literature on L3 that is concerned with higher level components of language related to cognitive writing processes is currently underdeveloped. This theoretical gap, as well as lack of empirical evidence, has motivated the current study.

Prior knowledge related to the use of sources in synthesis writing has been primarily based on think-aloud protocols (TAPs). This provides insight into why certain observed writing activities occur and how writers think of their own writing strategies. However, the observed reading and writing activities can be interrupted or even disrupted since writers cannot invest their full cognitive load into writing the tasks while verbalizing their thoughts simultaneously (Leijten et al., 2019). Moreover, Harwood (2009) raised concerns about the tendency of over-reporting when using retrospective measures: participants may not always be honest or reliable when self-reporting their cognitive processes as they may not recall every activity in the whole process. Recent advances in data collection, such as the advent of keystroke logging, e.g., Inputlog, have now enabled writing researchers to obtain fine-grained analyses of cognitive writing processes in an unintrusive manner (Leijten and Van Waes, 2013). As mentioned earlier, source use processes in the initial planning stage during a reading-to-write task may have a considerable impact on the whole writing process and the final product (Leijten et al., 2019; Vandermeulen et al., 2019). To date, few studies have investigated source use processes during synthesis writing from a process-oriented perspective. Recent studies like Doolan (2021) have explored various types of source integration and analyzed ideational units of students’ texts from an exclusively product-oriented perspective. Therefore, it would be methodologically enlightening if we could investigate source use processes using a process-focused approach, integrating keystroke logging into the research design. In a recent study by Leijten et al. (2017), the researchers examined source use processes during synthesis writing in a group of master’s students in a multilingual professional communication program of a Flemish university (*N* = 60). The postgraduate students wrote a text based on source texts in their L1 (Dutch). Using Inputlog – a keylogging tool, Leijten et al. (2017) were able to determine (a) the amount of time the students spent on consulting the sources, (b) the moments when the students consulted sources in the production process, (c) the sources which were consulted most frequently, and (d) the frequency of transitions the students made between the different source texts. This study indicated that the quality of written texts was related to how the students used the source texts, since it showed that relatively long initial reading time before writing, as well as frequent switches during writing contributed to the quality of final texts. Another notable finding was that both students’ source-use strategies and their text scores in L1 remained stable over the course of an academic year. In another study, Leijten et al. (2019) observed similar patterns for L2 writing processes when 280 Flemish students wrote texts in both Dutch and English. According to Leijten et al. (2019), there are three source use components that could describe source integration processes during L1 and L2 writing process: (a) initial reading time, (b) source interaction, and (c) variance of source use. Although Leijten et al. (2019) managed to identify certain variables and components that could explain source use processes in L1 and L2, they could not develop a coherent model that could explain synthesis writing processes in all language conditions due to the exclusion of an L3. This limitation has therefore motivated our current study. The intention to include an L3 in our study is motivated by two reasons. First, we aim to provide a theoretical model that could fully describe synthesis writing processes in L1, L2, and L3. To the best of our knowledge, most synthesis writing research has mainly focused on L1 and L2. By including an L3, we could provide a broader view of how L3 writing processes may differ from those of L1 and L2. Indeed, pedagogical research has shown that the way in which L1, L2, and L3 are acquired (i.e., intentional vs. accidental, implicit learning vs. explicit learning) has an influential impact on certain linguistic features of writers and speakers (Boers, 2017). However, little research has examined the effect of acquisition manner on how language users produce their language on a more general cognitive level, such as in a synthesis writing task. Second, in this modern age of international mobility, multilingualism is considered as a norm, rather than an exception. As synthesis writing is both a cognitively and socially demanding, we decide to take this factor into account in this study.

The current study is an extension study of Leijten et al. (2017, 2019). In this study, we focus on a within-subject comparison of synthesis writing across L1 (Dutch), L2 (English), and L3 (French). On a micro-linguistic level, several aspects of writing in L1, L2, and L3 may differ: L2 and L3 written texts may show less lexical diversity due to writers’ less developed lexical knowledge in non-native languages, for instance. However, on a higher cognitive level, writing in L1, L2, and L3 may not be very different. As mentioned earlier, knowledge about text structure in general, such as argumentative or coherence, seems to be integrated in L1, L2, and L3, forming a single system as the mastery of the writing competence in general increases, as illustrated by the Linguistic Interdependence Hypothesis (Kobayashi and Rinnert, 2008; Cummins, 2016). The way writers consult sources may not differ across languages even if they read different sources, either. Therefore, we aim to confirm whether the same variables can be used to describe source use processes during synthesis writing in L1, L2, and L3. In this study, we are interested in three components and seven variables that were obtained as a result of principal component analysis (PCA) in previous studies of Leijten et al. (2017, 2019), as shown in Table 1.

**TABLE 1:** Three components and seven variables that describe source use in synthesis writing (adapted from Leijten et al., 2019).

Components	Variables
Initial reading time	Proportion initial reading time vs. total reading time of sources Number of source switches during initial reading time (per min)
Source interaction	Number of sources (per min) Number of sources per content categories (per min) Number of switches between sources (per min)
Variance of source use	Relative time spent on text during first interval (out of 5) Variance of time spent on text during process

We first conduct a confirmatory factor analysis (CFA) on the L1, L2, and L3 dataset of 280 master’s students from the program of professional multilingual communication (*N* = 280). After that, we investigate which variables linked to source use correlate with text quality, using structural equation modeling (SEM) to determine such correlation. Finally, regarding the text quality and process data, we aim to determine whether the students’ text quality and source consultation strategies remain stable over an interval of 6 months. The research questions are as follows:

1. How do master’s students consult sources when writing integrated texts in L1, L2, and L3?
2. To what extent is there a relationship between source use approach and final text quality in L1, L2, and L3?
3. How stable is the individual text quality in L1, L2, and L3 at two moments of measurement, separated by an interval of 6 months?

## Materials and methods

### Participants

Our sample consisted of 280 Flemish master’s students who major in multilingual professional communication at the University of Antwerp (the gender ratio was 16% male-84% female, which is typical for this master’s program). The participants (age range: 20–34 years; mean age: 22 years and 10 months) all spoke and wrote Dutch as their native language (C2 level according to Common European Framework of Reference). These students completed communication courses in the master’s program both in their L1 (Dutch) and in one or more second/additional languages: a sub-group of students did some courses in English (*N* = 138, C1 level); the other students chose to study either in French, German, or Spanish. For these additional languages, the students were either tested or asked for B2 accreditation at the beginning of the program, which was an essential prerequisite for them to start the courses. By the end of their program, the students were expected to reach C1 level. Therefore, the participants who wrote in French for our study were all either tested or asked for B2 accreditation in French by the time of the experiment (*N* = 66, B2 level). This population was also recruited for the study of Leijten et al. (2019).

For this project, we collected data throughout three consecutive academic years: the first-year data which consisted of texts written only in Dutch (L1) by students from this master’s program (*N* = 60) were previously presented in an exploratory study (Leijten et al., 2017). All the 3-year data which included texts written in Dutch (L1) and English (L2) have been extensively used in another study (*N* = 280) (Leijten et al., 2019). In this study, we extended our analyses to include L1 (Dutch), L2 (English), and L3 (French), focusing on all language conditions. This particular population of advanced writers (i.e., master’s students from multilingual professional communication) was selected because of our intention to examine synthesis writing from a multilingual perspective. As these students speak and write multiple languages at an advanced level of proficiency (C1 in English and B2/C1 in French), we may evade lower-level writing problems related to language proficiency that hinder participants’ source use (i.e., participants having difficulty understanding sources correctly due to their lack of vocabulary). Moreover, these master’s students had prior experience in academic and business writing, a particular genre of writing that requires selecting primary ideas from sources, using multiple sources, and integrating information into target texts appropriately (Plakans and Gebril, 2013). By examining this particular writer population, we could focus on higher-order cognitive skills (e.g., synthesis skills), keeping the comparison between L1 and L2/L3 unaffected by language-related issues.

### Materials

The experiment took place both at the start and at the end of the master’s program. At two moments of measurement, the participants wrote two similar writing tasks in L1 (Dutch) and in L2 (English) or in L3 (French). At each moment, all students composed a text in L1 (Dutch) and a text either in L2 (English) or in L3 (French). So in total, each participant composed four texts for this experiment. On average, these texts varied between 200 and 250 words in length and were based on three external sources in different genres: a report (written in formal language), a web text (written in plain language), and a newspaper article (written in relatively formal language). The topics discussed in these source materials were controversial issues of the European Union, such as the protection of animal welfare, the use of renewable energy, the importance of humanitarian aid, and the impact of climate change. The source types (report, web text and newspaper article) for all topics did not differ considerably in terms of format, length, and complexity (e.g., the mean number of words per sentence was similar across materials and languages). Moreover, the participants were prompted to write for a specific group of audience: final-year students in secondary school. The diversity of content and language in the three source texts, along with the difference in the target audience of the sources and the new text, contributed to the task complexity for these advanced student writers. Table 2 indicates an overview of the material in Dutch, English, and French.

**TABLE 2:** Overview of the text length of the provided sources per theme and per language (mean number of words per sentence).

	Humanitarian aid	Energy	Animal welfare	Climate change
**Dutch**				
Report	316 (18.6)	321 (18.9)	321 (18.9)	323 (19.0)
Web text	237 (8.4)	229 (8.2)	227 (8.1)	227 (8.1)
Newspaper article	244 (15.3)	239 (15.0)	246 (15.4)	246 (15.4)
**English**				
Report	325 (19.1)	313 (18.4)	318 (18.7)	304 (19.0)
Web text	246 (8.8)	230 (8.9)	239 (8.9)	242 (8.9)
Newspaper article	251 (15.6)	242 (15.1)	250 (15.6)	244 (15.3)
**French**				
Report	346 (18.2)	344 (19.1)	335 (18.6)	333 (18.5)
Web text	253 (9.0)	240 (8.9)	259 (9.3)	274 (9.5)
Newspaper article	271 (16.4)	268 (16.8)	271 (16.9)	247 (16.5)

The participants’ writing activities were tracked with Inputlog 7 – a keylogging program that enables researchers to record all keystrokes, typed characters, mouse movements, and Microsoft Windows activities that take place within a Microsoft environment (Leijten and Van Waes, 2013; Leijten et al., 2014). Inputlog is a free open-access tool for writing research and can be easily downloaded from www.inputlog.net. All processes of a writer trying to produce a final document are logged with the corresponding time stamp. These detailed log files obtained from Inputlog served as the foundation for further analysis (see Leijten et al., 2019 for further details on the materials and task design).

### Procedure

The whole writing session consisted of a typing task and two writing tasks and took approximately 2 h in total. The session started with a warm-up typing task (Van Waes et al., 2015, 2021), which revealed the general typing speed of the students but simultaneously allowed the students to familiarize themselves with the computer room and the experimental environment. After the typing task, the participants were ready to start their writing tasks. At two moments of testing, the students were given 40 min to complete each writing task based on the three sources. The use of the Internet was not restricted. Our students were allowed to check for more general and linguistic knowledge or use Internet tools such as online dictionaries. Those who completed the tasks in less than 40 min were asked to write a brief description of any highlights and shortcomings of their master’s program in the L1. By doing so, we could ensure that our participants would not be distracted by their fellow students who finished writing sooner. After a short break, the students had another 40 min to complete the second writing task. At the end of the writing session, all participants completed a form which allowed us to process the collected data and informed them about their right to withdraw from the study at any time without any penalties or severe consequences.

### Text assessment

In order to assess the quality of the final texts, we opted for holistic pairwise comparisons, which was operated on the Digital Platform for Assessment of Competence (D-Pac, now Comproved^1^). This comparative assessment tool was previously used for Dutch and English texts in Leijten et al. (2017, 2019). The system presents two texts (A vs. B) and provides a choice between them by posing a question for the assessors: “Which text is the better elaboration of the task?” (van Daal et al., 2017). We chose this comparative assessment procedure because as shown in research, evaluators tend to be more reliable when they must compare the content of two texts than when they only grade an isolated text (Pollitt, 2012). Moreover, pairwise comparisons allow each text to be evaluated by multiple assessors, so the evaluation is a conglomeration of the assessors’ expertise (Pollitt, 2012).

For the D-Pac (now Comproved) assessment platform, we only selected written texts of students who participated into the writing sessions at both moments of measurement (in October and in April of the following year). For L1 Dutch, 68 students (*N* texts = 136) wrote texts at two measurement periods. The Dutch texts were evaluated independently of one another by 10 experienced evaluators, all of whom were affiliated with the master’s program in multilingual professional communication. We anonymized the texts and randomized the order in which all texts were evaluated for the 10 assessors. For L2 English, the texts of 35 students were marked (*N* texts = 70) by 4 experienced evaluators, who were all colleagues working with the Faculty of Arts at the University of Antwerp (see Leijten et al., 2019 for further details on how the Dutch and English texts were assessed). The same procedure was followed for French texts. For the L3 French, the text of 24 students were assessed (*N* texts = 48).

### Preparation and analysis of inputlog keylogging data

We first prepared the keystroke logging data (*N* = 280) in Inputlog version 7.1 for further data analysis. To perform a general data analysis on the writing process data, we used the summary analysis, the pause analysis, and the source analysis that were integrated in Inputlog (for further information, please consult Inputlog User’s Manual^2^).

In the initial analyses, we included initial reading time and writing activities that took place both within the main Microsoft Word document and in other sources, using the “summary analysis” (pause threshold of 2000 ms) and the “pause analysis” (pause threshold of 200 ms). First, we opted to use a general pause threshold of 2000 ms to determine the number of P-bursts (Van Waes et al., 2015). As shown in the literature, these bursts which revealed “a pronounced cognitive load during the process interruption” are usually related to planning and revision (Leijten et al., 2019). Second, we also opted for a pause threshold of 200 ms to consider the pauses which were linked to a lower cognitive load (Van Waes et al., 2015). These pauses allowed us to identify those process delays that are potentially not related to “the time it takes writers to move their fingers from one key to another” (Leijten et al., 2019). In the following steps, we conducted the “summary analysis,” as well as the pause analysis again, but included only the activities that occurred within the main document (excluding other Microsoft activities) (Leijten et al., 2019).

Subsequently, we performed a “source analysis” with Inputlog. We observed in total 3104 different focus events. A focus event is recorded in the log file when a writer opens a computer window. The Inputlog source analysis serves as the basis for a thorough recoding of the data: the main Microsoft Word document, the report, the web text, the newspaper article, and the other sources. The other sources consulted by the students were recoded and categorized into five categories. Table 3 provides insights into these additional external sources and tools that were consulted by the master’s students during the writing process.

**TABLE 3:** Five categories of the other consulted sources (also found in Leijten et al., 2019).

Content (19%)	Background information on the topics (e.g., report on the refugee crisis in a Flemish news program)
Language (64%)	Language: general (53%) (e.g., spelling of crisis vs. crises) Language: synonym (11%) (e.g., other word for regulate)
Other (12%)	Activities that cannot be classified as content, language, search, or task (e.g., opening Media Player)
Search (4%)	General searches online or on the computer (e.g., opening Google)
Task (1%)	Searches related to the task (e.g., what is a synthesis?)

### Confirmatory factor analysis and structural equation modeling

For the analyses of the current study, we mainly opted for CFA and SEM. Our analyses were performed in R (version 3.6.3 using the Lavaan library). We conducted these analyses to verify whether the component structure and the underlying variables that were found in the previous studies by Leijten et al. (2017, 2019) could account for the variance in source use processes across three different language conditions (Dutch as L1, English as L2, and French as L3). In addition, to measure the possible progress made by the students between the two moments of testing (beginning and end of the master’s program) and/or language conditions (L1, L2, and L3), we opted for a general linear model (GLM) (repeated measurements, multivariate analysis). We will briefly elaborate on the stepwise procedure that guided the analysis of the data.

With the CFA, we could investigate the relations among latent constructs and observed variables, obtained as a result of PCA reported in a previous study (Leijten et al., 2017), which focused on the data of the first cohort who wrote texts in Dutch (*N* = 60). In principle, CFA “explicitly tests *a priori* hypotheses about the relations” between observed variables (i.e., variables that could be directly observed or measured) and latent variables (i.e., components and dimensions that are not observable in a straightforward manner but could be “inferred from underlying, highly clustered variables”) (Leijten et al., 2019). In this study, the latent variables we mentioned were extracted from the exploratory PCA that was mentioned and elaborated in Leijten et al. (2017). After reducing the large number of variables generated by Inputlog to a neat and manageable set, Leijten et al. (2017) reported three components and seven variables that could be used to describe the variance in source use processes (Table 1).

In order to avoid sample dependence, Leijten et al. (2019) carried out the CFA on a reduced dataset which excluded the logfiles used in the PCA analysis in Leijten et al. (2017) and limited it to a single observation per participant. The final CFA model in this study, which consisted of three components and seven underlying variables, as shown in Table 1, was based on the model by Leijten et al. (2019). In this study, we replicated the CFA analysis, which was previously used for Dutch and English texts in the study of Leijten et al. (2019), on the data of French texts to generate a coherent model for source use processes across all language conditions. Using an iterative procedure, we strived to optimize the model by maintaining a good balance between “goodness-of-fit” (how appropriately the resulting model could be used to explain the observed data) and “parsimony” (limited collection of variables which considerably affect the resulting model). We also examined the fit indices described in the Result section. Followed by the Chi square test that indicates “the difference between the observed and the expected covariance” (Leijten et al., 2019), we used the comparative fit index (CFI) as a guideline to compare the target model with a so-called zero model (without any relations). According to the literature, a CFI score which is higher than 0.90 or even 0.95 is highly recommended to indicate a model with “good fit” (Hu and Bentler, 1999). Subsequently, we looked at the root mean square error of approximation (RMSEA), which is “the square root of the discrepancy between the sample covariance matrix and the model covariance matrix” (Leijten et al., 2019, p. 567). The literature recommends a value which is lower than 0.008 for a good fit and a value between 0.08 and 0.10 for “a moderate fit” (Hooper et al., 2008).

In our previous study (i.e., Leijten et al., 2019), we performed the CFA on the Dutch data (L1) and the English data (L2). In the current study, we applied the same model to the French data (L3), following the multiple group measurement partial invariance analysis. This partial invariance analysis allowed us to verify whether the variables and constructs used in the analysis remain comparable while allowing certain variables to be “partially non-invariant” across language conditions. We assessed the fit in a stepwise manner for the loadings, the intercepts, and the variables’ means after identifying and releasing the constraints on the most non-invariant variables in the components. Thanks to this evaluation, we could set the parameters equal or allow them to vary across languages in our final CFA model. We also used the same fit indices to identify the non-invariant variables and assess the measurement partial invariance. Finally, we ran the SEM to examine text quality in the analysis. This enabled us to map the relation between text quality and the three latent variables that were used to describe the source use characteristics and source interaction patterns of the participants in the CFA model. As mentioned earlier, this stepwise manner of data analysis, combining both the CFA and SEM, was also used in the study of Leijten et al. (2019). The CFA and SEM analyses in this study were replicated according to their results and models.

## Results

### General description of source use during synthesis writing

As mentioned before, the current study is an extension of the study by Leijten et al. (2019). Therefore, we briefly summarize some previous results regarding Dutch and English data in the study of Leijten et al. (2019) and include the data of French texts in our analyses and discussion. In general, the students had a maximum of 40 min to complete the writing task, using the three provided source texts. As reported in the study of Leijten et al. (2019), the average time the students needed for writing the tasks in Dutch (L1) was 32:48 min (SD = 7:07) and 35:01 min (SD = 6:16) in English (L2). They needed on average 36:98 min (SD = 4:68) for writing the tasks in French (L3). As shown in Table 4, on average, the students used 64.8% of their time for composing the text in Dutch (L1) and 35.1% for consulting the sources. Similarly, for English (L2), the students spent an average of 63% of their total time writing the main text and 37% reading the sources. The results are slightly different for French texts: 58.2% was spent on the main document and 41.8% on the sources when the students wrote their texts in French (L3). When comparing the proportion of writing time and source use time in L1, L2, and L3, we can see that the students tended to spend more time reading the sources in L3 (French), which could be a result of less developed language proficiency and the fact that French texts are longer to read than Dutch and English texts.

**TABLE 4:** Overview of the mean duration (in minutes) and the mean proportion of time spent consulting the provided sources and other sources involved.

	Text	Report	Web text	Newspaper article	Other (Table 3)
**Dutch**					
Mean duration (SD)	21:16 (5:45)	3:38 (1:39)	2:20 (1:19)	3:09 (1:27)	2:48 (2:50)
Percentage	64.1	10.9	7.1	9.5	8.4
**English**					
Mean duration (SD)	22:05 (5:11)	4:29 (2:14)	2:27 (1:71)	2:48 (1:21)	3.32 (2.40)
Percentage	62.4	12.7	7.0	7.9	10.0
**French**					
Mean duration (SD)	20.32 (3:94)	4:46 (1:95)	2:57 (1:50)	4:12 (1:52)	5:08 (3:44)
Percentage (%)	54.5	12.7	7.8	11.2	13.9

**FIGURE 1:**
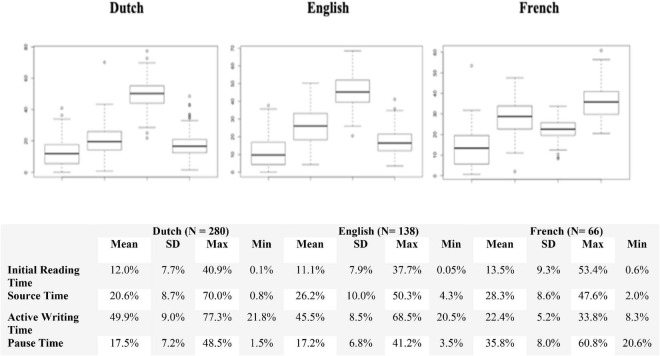
Overview of the initial reading time, source time, active writing time, and the pause time in percentages (100%) for Dutch (*N* = 280), English (*N* = 138), and French (*N* = 66).

In general, the students allocated 10% of their total time for getting acquainted with the given sources in the initial phrases. The rest of their time was spent on writing the main text, which accounted for rough 58–65% of their process time, of which nearly a quarter is taken up by pausing (pause threshold >2000 ms) (Figure 1).

As previously mentioned in “Materials and Tasks,” the participants composed their synthesis texts based on three source materials of different genres: a report, a web text, and a newspaper article. According to the results reported in Leijten et al. (2019), on average, the writers spent three and a half minutes on the report, two and a half minutes on the web text, and 3 min on the newspaper article when writing the Dutch texts. Approximately 3 min were used for consulting additional sources, which were related to content-related information or language-related information. For the English texts, the writers allocated on average four and a half minutes for the report – which is nearly a minute longer than in the Dutch writing task, two and a half minutes for the web text, and roughly 3 min for the newspaper article. A similar amount of time was spent consulting the given sources. Overall, the use of additional sources in English increased by about 19%. The only difference is the time spent on the text type: the students spent 16% longer of their time reading the report and spent 16% less of their time on the newspaper article. Another important thing to note is that the students spent about three and a half minutes on additional sources when writing the English texts, which is nearly a minute longer than they did in the Dutch texts. This difference is primarily related to a rise in the amount of time spent on language-related resources in the English texts, such as online dictionaries, thesauri, and grammar).

As for the French texts, the writers spent on average four and a half minutes on the report, nearly 3 min on the web text, and roughly 4 min on the newspaper article. Approximately 5 min were spent on additional sources. Overall, the writers spent considerably more time consulting content-related and language related information when writing the texts in French. This difference may be a result of variations in language proficiency as lexical knowledge and grammatical fluency tend to be least developed in L3, in comparison with L1 and L2.

As proposed in the study of Leijten et al. (2017), their model based on PCA accounted for 75% of the variance in source use processes of synthesis writing in L1 (Dutch). The model then served as the foundation for the CFA analysis, which was used for both the data of Dutch and English texts in the follow-up study by Leijten et al. (2019). The same procedure used in Leijten et al. (2019) is now extended to the data of French texts in the current study. By replicating the model and conducting the same analyses, we aim to verify whether the same model could be used to explain the variance to describe source use in synthesis writing in L1 (Dutch), L2 (English), and L3 (French).

### Confirmatory factor analysis of source use

The CFA model in this study, which consisted of the three components and the seven underlying variables of the PCA as described in Table 1, indicated a good fit on the one hand, but on the other hand also provided suggestions for future improvement. In the previous study of Leijten et al. (2019), the fit indices for this final CFA model illustrates a good fit of the model [Chi square for Dutch χ^2^(10) = 12.5; *p* = 0.253; CFI = 0.993 > 0.95; RMSEA = 0.043 < 0.10 and English χ^2^(12) = 22.4; *p* = 0.003; CFI = 0.959 > 0.95; RMSEA = 0.098 < 0.10]. Therefore, this model was used as the reference model for additional analyses on the French dataset of student texts [Chi square for French χ^2^(10) = 10.70; *p* = 0.381; CFI = 0.993 > 0.95; RMSEA = 0.040 < 0.10].

In order to measure the potential effect of the language used on the seven variables, we performed a multivariate analysis. As the normality test revealed that our data of students’ source use processes are not normally distributed, we opted for the Kruskal–Wallis test instead of the conventional one-way ANOVA. Interestingly, five out of the seven variables included in the model showed significant differences, more specifically “*Number of source switches between sources (per min)*,” “*Number of sources (per min)*,” “*Number of sources per main categories (per min)*,” “*Relative time spent on text during first interval (out of 5)*,” and “*Variance of time spent on text during process*.” In the previous study of Leijten et al. (2019), no significant difference was found between source use processes in L1 and L2, but in the current study, we found observable differences for some variables related source use processes between L1–L2 and L3. This is an indication that students seem to consult sources differently when composing their texts in L3 (French) than when writing in L1 (Dutch) and L2 (English). For example, the variable “Relative time spent on text during first interval (out of 5)” belonging to the “Variance of source use” component was found to differ significantly between L1–L2 and L3. This implies that students tend to spend less time composing the main text and longer time consulting external sources or checking language-related issues during the first interval when writing in their L3 (French) than they do when writing in L1 (Dutch) and L2 (English).

To verify whether the model can be used to describe and explain source use characteristics in synthesis writing in L1, L2, and L3, we opted for a multiple group measurement partial invariance analysis by allowing three variables, including “*Proportion initial reading time vs. total reading time of sources*,” “*Number of source switches during initial reading time (per min)*,” and “*Number of sources per content categories (per min)*” to be partially invariant across models. Such analysis indicated a strong/scalar invariance for all three language conditions (L1, L2, and L3): constrain item intercepts are equal across all languages in tandem with constrain factor loadings (Table 6). As demonstrated previously, only the fit for the variable means across languages found to be significant. This indicator has enabled us to fixate the model for all languages at both the loading and intercept level when setting up the final SEM model, involving quality scores (D-Pac scores), as mentioned in *Relationship Between Source Use Components and Text Quality*, which means we can use the model to describe source use processes in synthesis writing in L1 (Dutch), L2 (English), and L3 (French).

**TABLE 5:** Factor loadings for the CFA model in the Dutch, English, and French language condition, including descriptive values for the underlying variables.

	Dutch (*N* = 149)			English (*N* = 99)			French (*N* = 45)			Sign.
	CFA loading (Std. all)	*M*	SD	CFA loading (Std. all)	*M*	SD	CFA loading (Std. all)	*M*	SD	
**Initial reading time**										
Proportion initial reading time vs. total reading time of sources	0.912	0.294	0.204	0.995	0.296	0.205	0.852	0.219	0.157	0.830
Number of source switches during initial reading time (per min)	–0.778	4.236	5.034	–0.564	3.587	5.349	–0.927	3.775	3.661	0.279
Number of switches between sources (per min)	–0.248	4.580	2.387	–0.221	5.015	3.292	–0.039	5.441	1.766	**<0.01**
**Source interaction**										
Number of sources (per min)	1.095	0.296	0.179	0.889	0.394	0.216	1.780	0.497	0.266	**<0.001**
Number of sources per main categories (per min)	0.691	0.157	0.054	0.873	0.167	0.037	0.316	0.159	0.031	**<0.05**
Number of switches between sources (per min)	0.206	4.580	2.387	0.482	5.015	3.292	0.216	5.441	1.766	**<0.01**
**Variance of source use**										
Relative time spent on text during first interval (out of 5)	–0.952	0.274	0.201	–0.998	0.254	0.196	–0.628	0.152	0.138	**<0.001**
Variance of time spent on text during process	0.794	0.396	0.140	0.737	0.422	0.140	1.192	0.478	0.111	**<0.01**

Std. = standardized factor loadings; Sign. = significance (p value).

The bold values show significant p-values.

**TABLE 6:** Fit measures of the interlanguage measurement partial invariance analysis.

Measure^a^	Chi square	df	Significance (*p*)	CFI	RMSEA
Loadings fit	63.778			0.968	0.081
Intercepts fit	65.057	2	0.5275	0.969	0.078
Means fit	106.469	6	<0.01***	0.920	0.116

^a^Configural model did not converge.

*** = highly significant (p-value < 0.01).

### Stability of text quality at both measurement moments across three language conditions

As mentioned in section “Text Assessment” in the Methodology section, professional markers assessed the text quality written in three languages (Dutch, English, and French) using D-Pac platform that runs on the basis of pairwise comparisons. In order to examine the stability of text quality at two moments of measurement, we performed a GLM repeated measures on the *Z*-scores per language. Because of data loss due to technical issues and participant attrition (i.e., some participants did not write their texts at both measurement moments), we were able to obtain *Z*-scores from 68 participants for Dutch, 35 participants for English, and 24 participants for French (L3) (as shown in Table 7).

**TABLE 7:** Mean quality (*Z*-scores) per test moment in Dutch, English, and French.

	Moment 1 (October)	Moment 2 (April)	Significance
**Dutch (*N* = 68)**			
Mean quality score (SD)	−0.07 (1.1)	0.11 (0.9)	0.142
**English (*N* = 35)**			
Mean quality score (SD)	−0.21 (0.80)	0.22 (0.98)	0.062
**French (*N* = 24)**			
Mean quality score (SD)	0.07 (1.09)	−0.07 (0.91)	0.545

The results indicated that there is no improvement in the mean quality of the texts in three languages at the two measurement periods (an interval of 6 months). As reported in the study of Leijten et al. (2019), the average *Z*-score of the Dutch texts at measurement moment 1 (October) was −0.07 (SD = 1.07) and 0.11 (SD = 0.90) at measurement moment 2 (April). Also, the quality of the texts did not vary at the two measurement moments [*F*(1,67) = 2.204, *p* = 0.142, η*_*p*_*^2^ = 0.32] (Table 7). The quality of the texts written in English were also similar: the average *Z*-scores of the English texts was −0.21 (SD = 0.80) at the first measurement moment (October) and 0.21 (SD = 0.98) at the second measurement moment (April). Like the Dutch texts, there were no significant differences in terms of text quality of English texts at two measurement moments: *F*(1,34) = 3.728; *p* = 0.062; η*_*p*_*^2^ = 0.099 (Table 7). The same holds true for French in the current study because the average *Z*-score of the French texts at measurement moment 1 (October) was 0.07 (SD = 1.09) and −0.07 (SD = 0.91) at measurement moment 2 (April). Like the Dutch and English texts, the quality of the French texts did not vary between two moments of testing [*F*(1,23) = 0.377, *p* = 0.545, η*_*p*_*^2^ = 0.16] (Table 7).

Text quality across L1 (Dutch), L2 (English), and L3 (French) may remain stable between the two measurement moments in all languages, which suggests that the students’ writing ability and source use strategies did not change much over the period of an academic year (6 months). Although it is important to know that the quality of written texts did not differ at two test moments, we were also interested in how product quality relates to source use, whether high-quality texts are characterized by specific behaviors in source use, and whether this potential relationship remains identical across L1, L2, and L3. Therefore, it was necessary for us to run an SEM model on the texts to assess a potential relationship between the source components and text quality.

### Relationship between latent components of source use and final text quality

**FIGURE 2:**
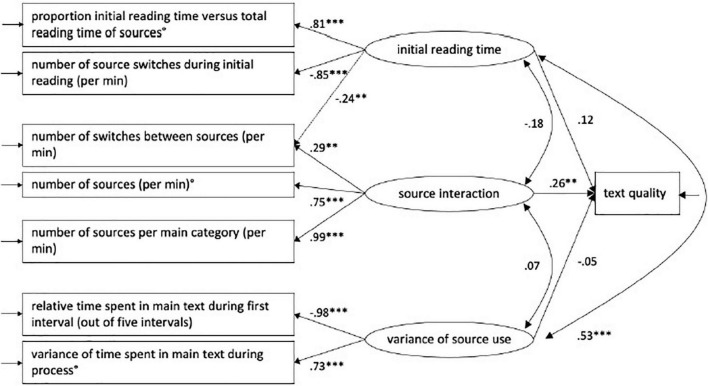
Structural equation modeling showing the effect of source interaction on text quality in L1 (Dutch) (Leijten et al., 2019). ** = mild to moderate correlations, *** = strong correlations.

**FIGURE 3:**
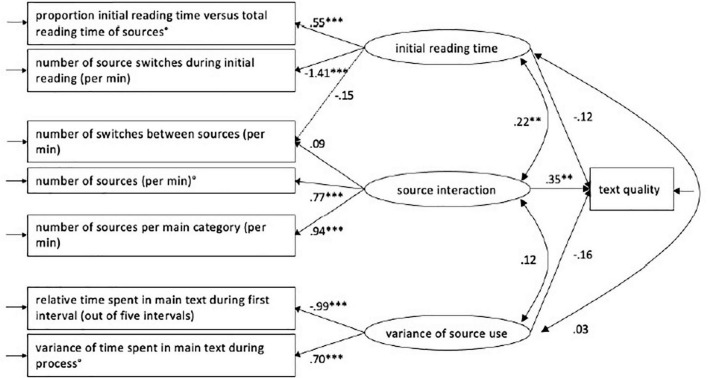
Structural equation modeling showing the effect of source interaction on text quality in L3 (French). ** = mild to moderate correlations, *** = strong correlations.

We conducted an SEM model based on the CFA model and included the text quality in order to evaluate the possible relationship between the source use components and quality of final texts (assessed *via* D-Pac scores), as described in section “Text Assessment.” The final models are shown in Figure 2 for Dutch and Figure 3 for French below:

As reported in the study of Leijten et al. (2019), in the L1 (Dutch) condition, text quality is significantly related to “Source use” with a path loading of 0.26 (Figure 2). This implies that when the standardized score for the “Source interaction” component increases with 1 SD, the standardized text quality score will increase with 0.26 SD. In the English L2 condition, this significant effect was not confirmed. However, in this study, the same effect of source use on text quality was found in the French L3 condition: text quality is found to be determined by “Source use” with a path loading of 0.35 (Figure 3). This indicates that when the standardized score for the “Source interaction” component increases with 1 SD, the standardized text quality score will increase with 0.35 SD (Table 8). In comparison with the L1 Dutch condition, the relation between “Text quality” and “Source interaction” in the L3 French condition is shown to be moderate but still significant.

**TABLE 8:** Standardized parameter estimates of the final SEM model.

	Estimate	Dutch Std. all	Sign.	Estimate	English Std. all	Sign.	Estimate	French Std. all	Sign.
Initial reading time	0.356	0.123	0.278	0.923	0.185	0.289	–0.466	–0.119	0.201
Source interaction	1.977	0.261	0.005	2.025	0.129	0.369	3.217	0.347	**0.049**
Variance source use	–0.476	–0.053	0.608	–0.734	–0.053	0.706	–2.305	–0.158	0.282

Std. = standardized factor loadings; Sign. = significance (p value).

The bold values show significant p-values.

## Discussion

### Summary of main findings in the current study

This study has shed light on how master’s students consulted and processed external sources in their writing processes and how source use processes influence text quality in L1, L2, and L3. In our study, the writing processes in L1 (Dutch) and L2 (English), or L3 (French) of master’s students from the 1-year program in Professional Communication were registered with Inputlog. In this section, we first summarize the main findings of our study and discuss them in relation to the contemporary literature in L1, L2, and L3 writing processes. We then proceed to highlighting how these results could serve as a solid foundation to explain synthesis writing processes in different languages, in terms of theoretical, methodology, and pedagogical contributions.

To begin, according to what we observed in CFA, there are certain variables and factors that can serve as indicators to describe and predict source use characteristics during the writing process of a reading-to-write task. These components were proved to be similar across three different languages – L1 (Dutch), L2 (English), and L3 (French): firstly, the initial reading time at the start of the writing process where writers consult sources and plan their writing; secondly, the interaction writers make between the sources; and thirdly, the degree to which source use varies throughout different phases of the writing process. We are convinced that these well-defined components are sufficient for describing synthesis writing processes in future research. When comparing the underlying processes, we observed both similarities and differences across three different language conditions, especially in terms of the source interaction component. Overall, the student writers tended to consult a wider range of sources per minute in English (L2) and in French (L3) than in Dutch (L1). For the context of this study, we attribute this difference to a less developed system of lexical knowledge and grammatical competence in second language and third language, which leads writers to rely on external sources where they can extract vocabulary and grammar input for producing their target texts. On a higher cognitive level, the writing processes of the students across different languages are very similar and can be described using the same variables; variation will primarily occur in the use of additional sources due to linguistic issues (e.g., looking up words in the online dictionary).

In relation to the link between text quality and source use, we observed no improvement in text quality in L1, L2, and L3 between the two moments of measurement, which were separated by a 6-month interval. However, the participants in this study received extensive instruction during the 1-year master’s program in Professional Communication to acquire the necessary skills for writing from sources in L1 (Dutch), L2 (English), and L3 (French). They were trained to use their high-level mastery of general writing skills, which were acquired in academic writing assignments during their undergraduate studies in (applied) linguistics. In a similar vein, Tigchelaar and Polio (2017) did not observe any progress in students’ writing performance either: their semester-long English as a second language academic writing course failed in turning their participants into “autonomous writers, able to self-monitor linguistic errors and edit their own work.” Taken together, these results serve as concrete evidence that academic writing instruction may not always be effective in helping students to improve their writing competence and call for the integration of specific strategies in teaching writing from sources in L1, L2, and L3. Additionally, *via* the SEM, we found that text quality and source use were correlated in the Dutch condition (L1) and the French condition (L3), but not in English (L2). This contrast is an interesting finding that may intrigue future research into multilingualism and synthesis writing processes (see below in section “Limitations and Directions for Future Research”).

As mentioned in “*Review of the Literature*,” there is currently a lack of process-oriented research on source use in writing, which has posed a challenge for us because we do not have a solid existing literature with which we could explain and contrast our findings. Our biggest contribution in this study was to pinpoint three components (initial reading time, source interaction, and variance of source use) that can be used to describe synthesis writing processes in L1, L2, and L3. We also provide insights into how CFA and SEM can be used to examine the relation between these components of writing and writing performance. To begin, among these components, “initial reading time” component may be related to studies that focus on planning phases of writing. Longer and more attentive initial reading time may lead to higher text quality but might result in longer time spent on writing tasks (Ellis and Yuan, 2004; Johnson et al., 2012). In addition, careful planning in the initial phases has a positive impact on writing fluency and linguistic diversity, such as production rate of words or sentences per minute or more diverse vocabulary use (De Smet et al., 2014; Limpo and Alves, 2018). As suggested in theoretical constructs of writing research, initial planning and reading is a vital component in writing (Bereiter and Scardamalia, 1987; Hayes and Nash, 1996; Kellogg, 2008), although we are unable to confirm the relation between initial reading time and text quality in the current study. Simultaneously, our study has suggested that source use processes alone could not explain text quality, as synthesis writing is a cognitively complex process that involves the dynamic interplay between reading and writing. Therefore, text-productive or source integration strategies may also intervene with text quality. Undeniably, to write a synthesis text successfully, writers need to not only read and understand the source materials but also know how to integrate sources into their main text effectively (see below in section “Limitations and Directions for Future Research”).

Moreover, all the three components we explored here in our study are directly linked to the temporal distribution of the cognitive writing process. As writing is a cognitively demanding process, it is important to acknowledge the importance of the temporal distribution of the processes (Alves et al., 2008; Roca de Larios et al., 2008; Van Waes and Leijten, 2015; Xu and Qi, 2017; Barkaoui, 2019). The temporal process of source use in writing has been formulated mostly on theoretical grounds, and empirical research regarding this is still lacking, especially on large datasets and in different languages, as in our study. Therefore, we suggest that the temporal distribution of the writing processes by our masters’ students in the current study has reflected the theoretical construct of writing as a process that includes a number of components, such as planning, translating, composing, and revising. As Beauvais et al. (2011) mentioned in their article, the cognitive effort and distribution of writing processes may affect the characteristics of final texts. We therefore argue that the temporal distribution of writing process is central to the quality of written texts.

Finally, as we compare writing processes of master students in L1 (Dutch), L2 (English), and L3 (French), we are able to revisit some respects of the Linguistic Interdependence Hypothesis by Cummins (2016), using a process perspective instead of a product lense: language users may form a unified system in the mind once they have reached a certain level of proficiency; regardless of differences in languages used, similarities may be observed in cognitively demanding tasks, especially those that require high order skills, such as reading and writing. In this study, we observed that the temporal distribution across three languages is relatively similar in three language conditions, using a large dataset. The quality of written texts remains stable at the beginning and at the end of the academic year, suggesting that neither writing strategies nor internal cognitive processes that underlie synthesis writing have undergone any changes over an interval of 6 months. CFA and SEM analyses also revealed similar patterns in the relation between the components that regulate the writing process, temporal distribution, and quality of final texts. None of the components among initial reading time, source interaction, or source use variance were related to text quality in L2 (English), except in L1 (Dutch) and L3 (French), where source interaction was related to text quality. However, as we mentioned, this contrast is obscure and may require further research.

### Novelties of the current study

In general, our study has opened doors for future research into the effect of multilingualism on synthesis writing processes and called for an update of pedagogical intervention strategies for training multilingual students to integrate sources into their texts since quality did not improve without specific training on source use. By including an L3 in the current study, in addition to L1 and L2 as in the study of Leijten et al. (2019), we have provided a theoretical model to fully describe synthesis writing processes in L1, L2, and L3. As mentioned previously, there is no research that has attempted to provide such model. As Leijten et al. (2019) provided some data that could explain variance in source use processes in synthesis writing only in L1 and L2, there was a strong need for us to include an L3 in the current study to provide a coherent model that could explain synthesis writing in all language conditions. To that end, we have added values to the current literature in synthesis writing research by providing insights into how L1, L2, and L3 writing processes may remain similar or differ on theoretical, methodological, and pedagogical grounds.

Regarding the theoretical contributions, we have succeeded in pinpointing the components and variables that could be used to describe and explain source use in cognitive writing process in different languages. These components and variables may become transferrable to any writing studies that aim to examine synthesis writing from a process perspective. Most importantly, we have illustrated that the initial amount of time reading external sources at the beginning of the writing process is essential. We can relate these findings to a number of planning studies in L1 and L2 (Ellis and Yuan, 2004; Johnson et al., 2012; De Smet et al., 2014; Limpo and Alves, 2018). In addition, we have contributed to the synthesis writing literature by investigating source use process in third language writing, which has been an unexplored area in writing research to date. This exploration of third language writing has revealed that the cognitive processes that underlie synthesis writing are similar across different languages, regardless of language proficiency. Differences in relation to linguistic proficiency may be observed at low-order levels of language, such as grammatical competence or vocabulary use, while leaving high-order levels of cognition, including planning, and composing the writing task unaffected (Cummins, 2016). Therefore, the result of the current study argues in support of the Linguistic Interdependence Hypothesis: on a higher cognitive level, synthesis writing processes are relatively similar across different languages; these processes are also observable and describable by using the same variables as reported in our study. However, it is important to note that differences in language proficiency may lead to minor differences in how writers consult sources in different languages. For example, in our study, the students consult a greater number of sources in L2 and L3, in comparison with L1.

At the methodological level, we have illustrated that it is important for writing researchers to carefully extract the relevant variables that are derived from the keystroke logging data collection. We have also pinpointed the components and variables that could describe the temporal distribution of writing process, including both variables that are related to reading process and variables that are related to writing in the synthesis writing process. These variables may be transferred for use in a vast array of writing process studies, which makes results from various studies comparable and coherent for meta-analyses or replication in different languages (Van Waes et al., 2015; Baaijen and Galbraith, 2018). For future research on source use during the writing process, we have also pioneered in the use of Inputlog to extract and examine all the cognitive variables that intervene with writing behaviors and text quality. These cognitive variables do not seem to change in various languages, so future writing researchers may investigate synthesis writing processes using the same variables, even in different languages other than English.

From a pedagogical perspective, we recommend that writing instructors should teach synthesis writing using a process perspective and practice different techniques for teaching different components of the writing process. Writing instructors should help students to realize the importance of deliberate fragmentation of the cognitive writing process, starting from planning, reading, writing, and revising the written texts. According to Doolan and Fitzsimmons-Doolan (2016), a sequential approach of teaching synthesis writing may have a more beneficial impact on writing performance of students than summary instruction, which exclusively focused on the summary as the ultimate goal. They propose to incorporate summarizing and paraphrasing techniques into larger written assignments, such as essays, in which the interplay between independent and integrated writing is important, since paraphrasing and summarizing strategies are often practiced in isolated writing contexts. Such instruction may enable student writers to envision various characteristics and different writing strategies that exist in synthesis writing process.

### Limitations and directions for future research

This study investigates the use of external sources among master’s students when writing synthesis writing tasks in different languages from a technical process perspective, using Inputlog. Unlike writing studies using TAPs or stimulated recalls (López-Serrano et al., 2019; Révész et al., 2019), this approach does not enable us to understand exactly what the students have read in the sources, how they have decided to adopt certain strategies, nor how they have integrated the selected information into their synthesis texts. Due to a completely quantitative research approach, we understand that our study cannot overcome some limitations and pitfalls in terms of methodology and reliability of the findings. However, we consider these limitations as fertile grounds and additional opportunities for future research. In this section, we highlight some notable limitations and suggest some improvements for future synthesis writing research.

An integrated writing task is considerably different from a writing-only task because integrated writing requires writers to draw on several source materials before arriving at final texts (Leijten et al., 2019; Vandermeulen et al., 2019). Consequently, writers do not write only from prior experience nor subjective opinions. One prerequisite in composing a reading-to-write task is that writers can comprehend the information represented in source materials. According to Cumming (2013), one primary issue with integrated writing tasks is that the writing behavior is affected not only by variables that are exclusive to the writing process, such as source consultation, planning, writing, and revisions, but also by factors that include the previous understanding of the source material (e.g., reading skills, background knowledge, area of interest, or expertise, etc.). It is important for follow-up research to take it into consideration. In this study, we did not measure the students’ reading competences nor their prior knowledge of the various topics, which led to a very diverse student sample. By considering these aspects, we could try to determine whether students read longer because they have difficulty understanding source materials, or whether they deliberately read slowly in order to obtain the information as accurately as possible. As Plakans (2009b) figured out in his research, L2 writers with low proficiency of reading abilities may have trouble comprehending source materials, which hinders their ability to compose texts effectively. Apart from linguistic proficiency and background knowledge, several cognitive processes, such as working memory capacities, may come into play. Individuals with weaker working memory capacities may read texts at a slower speed as a way to record information because they could not process larger chunks of texts (Medimorec and Risko, 2016; Zabihi, 2018). These components remain largely unexplored in the current writing literature, hence the potential of follow-up research.

Future intervention studies are also needed to map out how students should allocate their time to different activities during the writing process. After the current study, it remains an open question of whether it would be useful for student writers to focus on certain activities in certain intervals during the process, such as first reading of the sources (using dictionaries for only comprehension problems), then planning the text and finally writing the text (using sources for synonyms, looking up equivalents in L2/L3) (Vangehuchten et al., 2018). As indicated in prior research that examines synthesis writing from a process perspective (Leijten et al., 2017, 2019; Vandermeulen et al., 2019), it remains unknown how the distribution of specific activities in certain temporal fragments may influence the quality of final texts. For example, we do not know whether a considerable amount of time reading the source materials intensively at the beginning will have a beneficial impact of the quality of final texts. Such intervention studies using a process-oriented perspective may have meaningful contributions for pedagogical matters in second language writing training.

As indicated *via* the results of Leijten et al. (2017, 2019), and the current study, we found a partial correlation between source interaction and quality, but such relationship only exists in Dutch (L1); the text quality improved in parallel with more variation in source use. The absence of such relation in English (L2) can be explained in terms of two reasons. To begin, a lack of variation in the English and French sources might be the reason why no correlation was found between source interaction and quality in English as L2 and in French as L3. For one writer to either repeatedly consult the same sources or consult a vast array of sources can exert profound effects on the richness of information, syntactic structures, and lexical diversity. Additionally, the absence of a relation between source interaction and text quality in L2 might be a consequence of the differences in the size of the L1 and L2/L3 datasets. In general, there was a great amount of within-subject variation in the synthesis writing data. In the smaller L2 dataset, within-subject variation in source use may have obscured impacts of source use on text quality. Therefore, in future research, we will strive to collect a larger amount of L2/L3 data to address this limitation. Finally, the familiarity with using sources in L1 may add to the absence of such relation between source interaction and text quality: the participants may have simply repeated the same procedure of consulting sources and writing the texts in a different language that is not native. We may handle this limitation by asking different groups of students to write different texts in different languages in between-subject study design for comparison purposes.

This study has also provided several insights that may shape directions for further research in source use and synthesis writing. On the one hand, the added value of keystroke logging in writing research, such as Inputlog, is that we could accommodate a large group of students in order to obtain very rich writing process data that can be complementary to the more qualitative studies on the basis of TAPs (McGinley, 1992; Lenski and Johns, 1997; Plakans, 2008; Mateos et al., 2014; Chan, 2017). On the other hand, a mixed-method study with both quantitative and qualitative, in which participants are asked to read sources and write texts in an eye-tracking lab with their keystrokes being registered, would provide useful additional insights if they were also asked to verbalize their inner thoughts, consultation strategies, and composing processes in subsequent think-aloud or semi-structured interviews, as what De Smet et al. (2018) did in their study.

Moreover, follow-up research could strive to compare similarities and differences between the sources and the target texts, using plagiarism softwares or annotation tools. These so-called softwares and tools, such as TurnItIn, SafeAssign, or Scribbr could identify identical passages and directs them to the source material (Nguyen and Buckingham, 2019). The existence of several computer algorithms for automated text comparison would also generate richer analyses of written texts and facilitate corpus writing research, as these algorithms have successfully been applicable in journalistic science (Boumans and Trilling, 2016) and computer linguistics (Lin et al., 2014; Kim and Crossley, 2018). Computer algorithms have also enabled writing researchers to investigate lexical similarities between writing products and related source materials (Plakans and Gebril, 2013; Gebril and Plakans, 2016; Kim and Crossley, 2018; Neumann et al., 2019). Writers use sources not only for content but also for language and text organizational support (Plakans, 2009a; Plakans and Gebril, 2012; Ye and Ren, 2019). These traces of “textual borrowing” may not always be detectable in the final products, but may be visible in the writing process, which enables us to distinguish between extrinsic plagiarism – plagiaristic patterns that are distinguishable in the products and intrinsic plagiarism – plagiaristic patterns that could only be traceable in the writing process. Follow-up research that provides insights into these various perspectives on text similarities should adopt a process analysis of copy-paste behavior and pose a critical question – At what stage do students tend to copy passages from the source materials and how do they edit passages to integrate them into a coherent text? This consideration would allow us to envision a process and product approach for synthesis writing research in an innovative and enriching manner (Cislaru, 2015; Leijten et al., 2015, 2019).

Most importantly, we see a potential intervention study with a specific process-oriented pedagogical approach that incorporates Inputlog into the provision of process feedback and writing process regulation. Currently, Inputlog has only been used as a keystroke logging tool for writing research, and its application has not been extended to classroom use for second language writing teachers because of its technical complexities. The fine-grained analyses that Inputlog could provide might serve as a powerful tool for writing instructors to understand more deeply about their students’ synthesis writing processes that could be not clearly understood by just looking at the end products. The application of Inputlog into providing writing feedback for students has the potential to revolutionize the way we teach and remedy writing in second language classroom. In this study, we did not observe any improvements in writing efficacies of student writers in our cohort, regardless of measurement moments or writing languages (L1, L2, or L3). As this was a quantitative study that samples a large group of students, this does not mean no students made actual progress throughout the academic year. Some individuals may have improved their writing capacities, even to a very small extent. However, as mentioned before, synthesis writing is a cognitively demanding task that may go beyond only micro-levels of linguistic properties, such as lexical resources or grammatical competency. Improvements in low-level linguistic properties alone could not contribute to enhanced writing efficacy in general.

Finally, it is important to note that individual differences, for instance, learning style, writing belief, or self-regulation are essential for writing improvement. We could relate these features to some major findings of Baaijen et al. (2014), Limpo and Alves (2017), and Baaijen and Galbraith (2018), all of which focused on writing attitudes, the importance of goal achievement, and self-efficacy, respectively. According to Baaijen and Galbraith (2018), transactional beliefs in writing, which refers to the preference for a top-down writing strategy or a bottom-up writing strategy, and transmissional beliefs, which implies writers’ perception of the content to be written about, may influence text quality differently. According to the same authors, these beliefs may result in diverging effects on final text quality, the number and type of revisions made, and the degree to which writers develop their understanding (Baaijen and Galbraith, 2018). Limpo and Alves (2018) also confirmed the role of personal characteristics (e.g., goal achievement, self-efficacy) in forming the relation between writing strategies and writing efficacy. Taken together, these findings have suggested the importance of writers’ personal characteristics when examining the effect of writing sub-processes or writing strategies on writing performance. We hereby propose that it is important for writing researchers to consider personal characteristics as mediating variables when studying writing quality in follow-up research. Also, these writing attitudes may differ in multilingual settings, and writers’ profiles and perception may differ in different languages (L1, L2, or L3) because of variation in linguistic proficiency or working memory load. These variables are crucial in future writing research.

## Conclusion

In conclusion, this quantitative study into source use approach and synthesis writing from a multilingual perspective has provided several theoretical, pedagogical, and methodological insights, using Inputlog as a foundation. By investigating the writing processes across three different language conditions (L1, L2, and L3), we have highlighted three relevant components that could describe source use processes of master’s students in professional communication, which include initial reading time, source interaction, and variance of source use. As shown in the current study, source use and text quality are correlated, but only in L1 (Dutch) and French (L3). This relation was not found in English (L2) after we carried out CFA and SEM on a large dataset of students’ writing processes in different languages. These results have opened doors for many research potentials regarding synthesis writing processes, intervention research, intra-personal characteristics in writing, or the impact of multilingualism on synthesis writing. This study provides concrete evidence that source use processes alone cannot fully explain final text quality, and future writing research needs to investigate other factors, such as text-productive or source integration strategies and working memory.

## Data availability statement

The data analyzed in this study is subject to the following licenses/restrictions: The authors can provide datasets upon requests. Requests to access these datasets should be directed to LC, luan.chau@uantwerpen.be.

## Ethics statement

The studies involving human participants were reviewed and approved by the Ethics Approval Board, Department of Management, University of Antwerp, Antwerp, Belgium. The patients/participants provided their written informed consent to participate in this study.

## Author contributions

LC conducted the literature searches, provided the summaries of previous research studies, conducted the statistical analysis under supervision of ML, SB, and LV, and wrote the first and finalized draft of the whole manuscript. ML, SB, and LV designed the study, performed the data collection, and revised the manuscript. All authors contributed to and have approved the final manuscript.

## Conflict of interest

The authors declare that the research was conducted in the absence of any commercial or financial relationships that could be construed as a potential conflict of interest.

## Publisher’s note

All claims expressed in this article are solely those of the authors and do not necessarily represent those of their affiliated organizations, or those of the publisher, the editors and the reviewers. Any product that may be evaluated in this article, or claim that may be made by its manufacturer, is not guaranteed or endorsed by the publisher.

## Footnotes

1
https://comproved.com

2
www.inputlog.net
